# Uncommon Presentation of Solitary Plasmacytoma in the Nasal Cavity: Diagnostic and Therapeutic Challenges

**DOI:** 10.7759/cureus.65863

**Published:** 2024-07-31

**Authors:** Mohammed Amine Guerrouaz, Samah Tahri, Hanane Mansouri, Soufiane Berhili, Mohamed Moukhlissi, Loubna Mezouar

**Affiliations:** 1 Department of Radiation Therapy, Centre Hospitalier Universitaire Mohammed VI, Oujda, MAR; 2 Internal Medicine and Immunohematology and Cellular Therapy Laboratory, Mohammed First University, Faculty of Medicine and Pharmacy, Oujda, MAR

**Keywords:** solitary plasmacytoma, bone marrow, chemotherapy, radiation therapy, multiple myeloma

## Abstract

Extramedullary solitary plasmacytoma (SP) is an uncommon tumor and is even rare in the head and neck locations. Here, we report the case of an 82-year-old man admitted to our department for the management of nasal cavity SP. Radiological investigation showed a locally advanced tumor making the patient a non-candidate for surgery. The patient had undergone radiotherapy alone to a total dose of 50 Gy, with 2 Gy per fraction five days a week. After a follow-up of nine months, the tumor recurred, and the patient was managed in the internal medicine department. He received palliative chemotherapy with the cyclophosphamide, dexamethasone, and thalidomide protocol which resulted in a good response. This case illustrates the diagnostic challenges and treatment complexities of SP, particularly in rare locations such as the nasal cavity.

## Introduction

Solitary plasmacytoma (SP) is defined as a localized concentration of neoplastic monoclonal plasma cells. Among these, nasal cavity plasmacytomas are exceptionally uncommon [1]. Defined by stringent criteria set by the International Myeloma Working Group [2], SP poses challenges in diagnosis and management, with potential progression to multiple myeloma in a subset of cases. This article provides a concise overview of SP, with a focus on head and neck involvement. We explore clinical presentations, diagnostic markers, and management strategies, shedding light on the distinctive aspects of SP in the head and neck region.

## Case presentation

**Figure 1:**
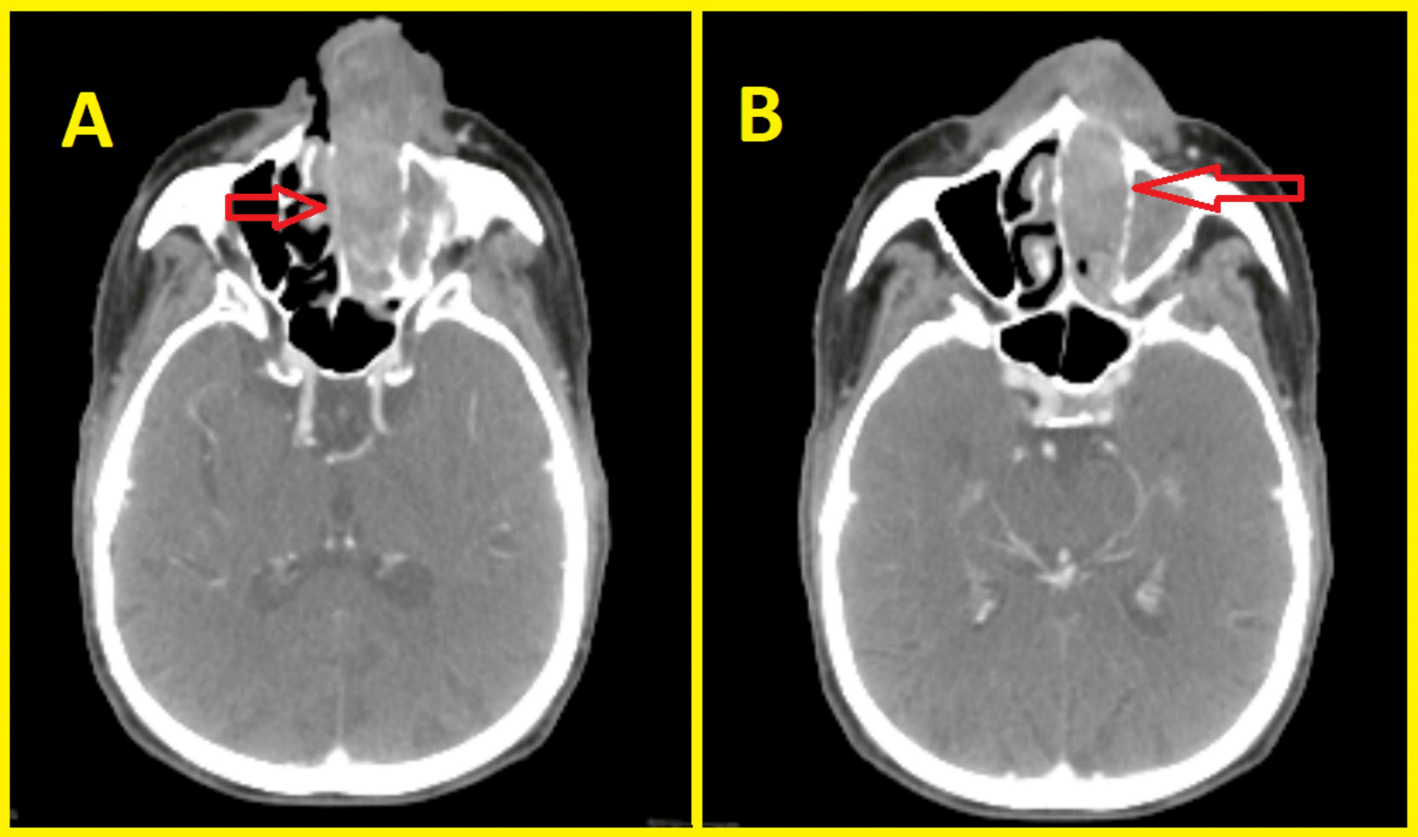
CT scan showing an expansive mass occupying the left nasal cavity and extending to the nasal septum (A), the maxillary sinus, the nostril anteriorly, and the choanae posteriorly (B) (red arrows).

An 82-year-old man was admitted to the otorhinolaryngology department for epistaxis and progressive bilateral nasal obstruction during the last year neglected by the patient. This symptomatology was marked by an increase in the nose volume more marked on the left side with protrusion of a nostril mass. The clinical examination was unremarkable and did not show any signs apart from those mentioned above. Subsequently, a CT scan with contrast of the skull base to clavicle was obtained and showed an expansive mass occupying the left nasal cavity measuring 57 × 24 mm and extending to the nostril anteriorly to the choanae posteriorly. The tumor mass destructed the medial wall of the left maxillary sinus laterally and invaded the latter, and it also infiltrated the nasal septum medially (Figure 1).

**Figure 2:**
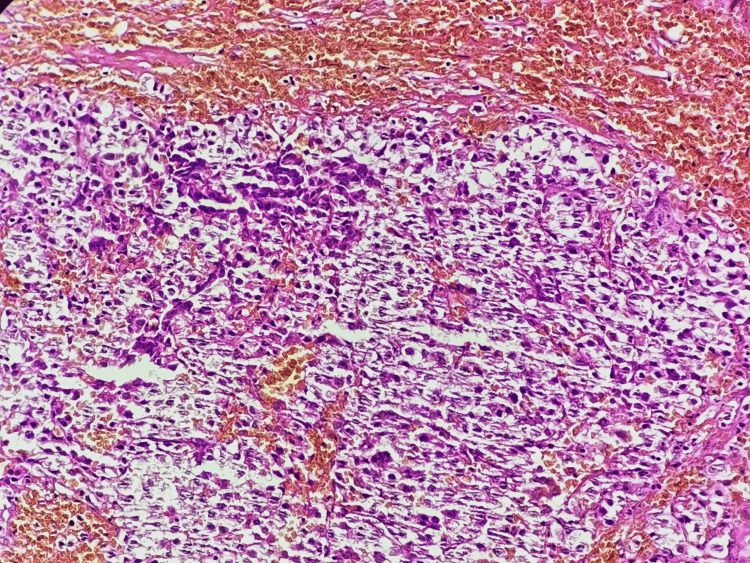
Microphotography showing infiltration by sheets of monomorphic cells showing a pseudo-cohesive pattern (hematoxylin and eosin; 200×).

**Figure 3:**
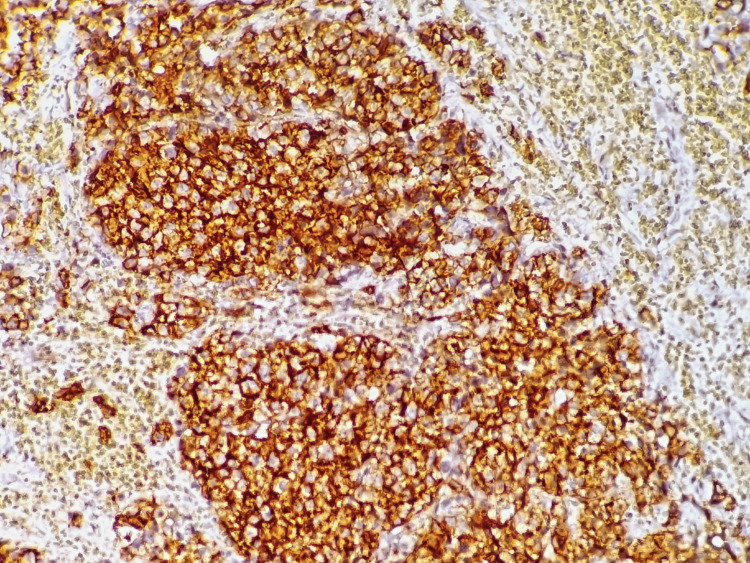
Microphotography showing expression of CD138 by neoplastic cells.

**Figure 4:**
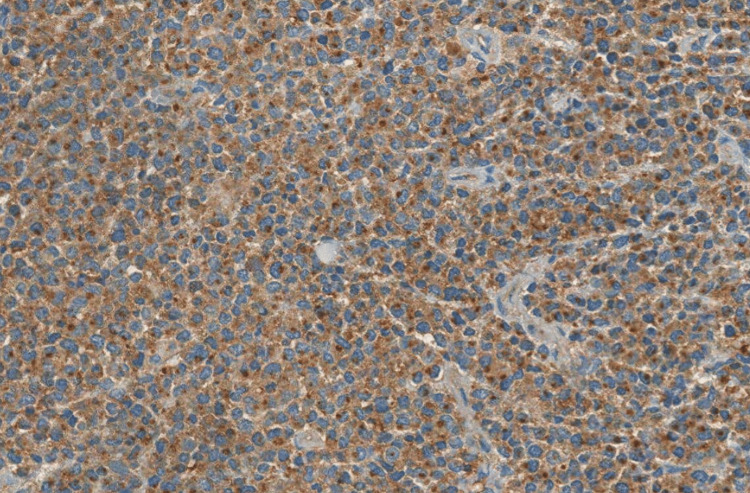
Microphotography showing expression of lambda light chains by neoplastic cells.

**Figure 5:**
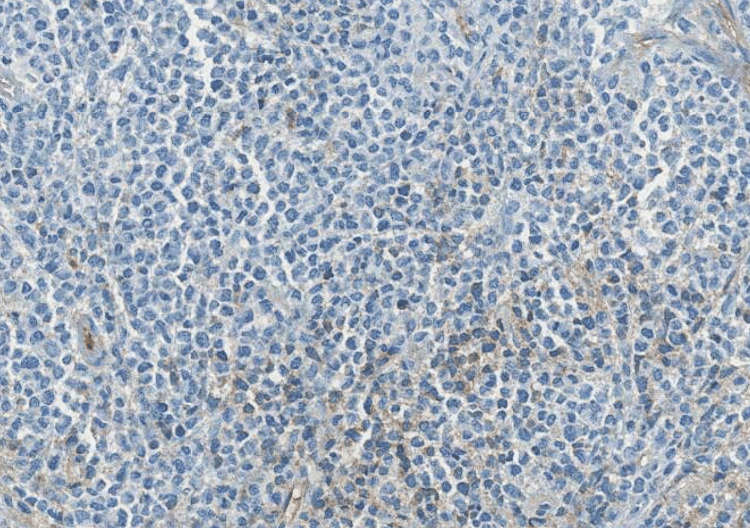
Microphotography showing lack of expression of kappa light chains by neoplastic cells.

There was no lymphadenopathy on the neck CT. The patient underwent a biopsy, and the histological examination showed tumor cells with hyperchromatic eccentric nuclei, perinuclear hof, and eosinophilic cytoplasm. On immunohistochemical analysis, the cells were positive for CD138 and CD79a, with a lambda expression, and negative for CD20, CD3, chromogranin A, synaptophysin, CKAE1/AE3, CD30, human herpesvirus 8, and Epstein-Barr virus, confirming the diagnosis of plasmacytoma (Figures 2-5). Afterward, a chest-abdomen-pelvis CT scan was performed and eliminated any other localization.

**Figure 6:**
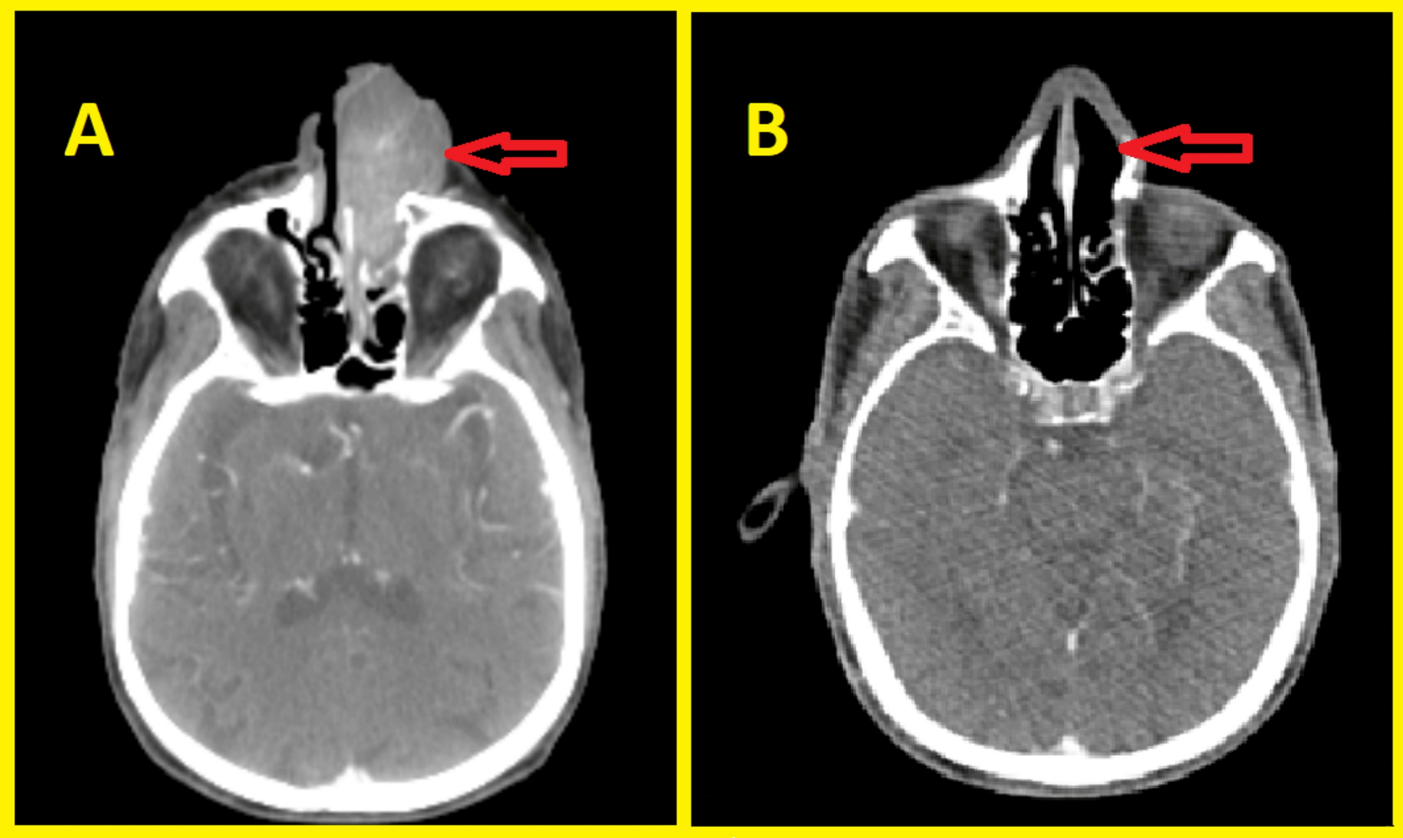
CT scan showing the nasal process before (A) and nine months after the completion of radiation therapy (B) (red arrows).

**Figure 7:**
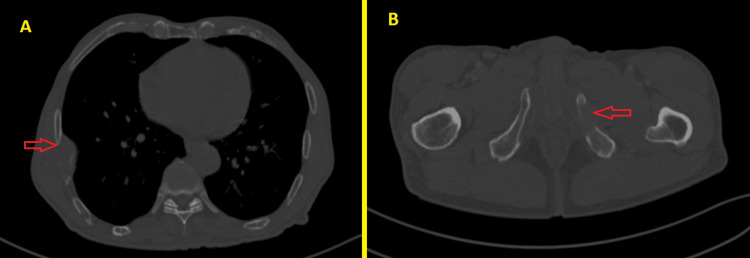
Thoracic-abdomen-pelvis CT scan showing the right rib (A) and left pubic infiltration (B) (red arrows).

The patient underwent other investigations including complete blood count, protein electrophoresis, urine protein electrophoresis, and bone marrow examination without any abnormality which affirmed the diagnosis of SP and excluded multiple myeloma (MM). The patient refused surgery after learning about the post-surgical complications. Hence, he was treated with radiation therapy alone. The target volume consisted of the delineation of the gross tumor volume plus a margin of 2 cm to encompass the microscopic disease with a 3 mm margin for the planning tumor volume. The total radiation dose was 50 Gy, with 2 Gy per fraction five days a week. Nine months after completion of radiation therapy, a CT scan showed a complete response of nasal tumor and the presence of a lymph node on the neck (Figure 6). Hence, an excision of the lymph node was performed and confirmed regional recurrence. He was referred to the internal medicine department after a CT scan showed more bone lesions (ribs and pubic bone) (Figure 7).

The patient is currently alive and undergoing palliative chemotherapy with cyclophosphamide, dexamethasone, and thalidomide with a partial response.

## Discussion

SP is a rare tumor, and nasal cavity plasmacytoma is a rare location, with head and neck SP accounting for fewer than 1% of all plasmacytomas [1]. It is defined as a plasma cell disorder characterized by a localized mass of neoplastic monoclonal plasma cells [2].

SP was defined by the International Myeloma Working Group by the following criteria: biopsy-proven solitary lesion of the bone or soft tissue with evidence of clonal plasma cells; normal bone marrow or clonal bone marrow plasma cells <10%; no other skeletal location with normal MRI (or CT) of the spine and pelvis; and the absence of end-organ damage. In the absence of bone marrow involvement, the evolution to MM is about 10% within three years, and in the presence of clonal bone marrow plasma cells <10%, the risk of progression increases to 60% for bone location and 20% for soft tissue location [2]. According to a retrospective Surveillance, Epidemiology, and End Results database study, the most frequent bone location was the axial skeletal, accounting for more than 80%, and the most common extramedullary site was the upper airway tract (12%) [3]. Men are more often affected by head and neck extramedullary plasmocytoma (EMP) [4]. The clinical presentation of SP is heterogeneous and depends on tumor location.

The histological diagnosis after lesion biopsy or fine-needle aspiration shows tumor plasma cells with eccentric nuclei and perinuclear hof. On immunohistochemical analysis, the cells often express CD138, CD79a, epithelial membrane antigen, and kappa or lambda markers and are negative for CD20 and CD45, as in our case [5]. Furthermore, other complementary investigations should be done such as complete blood count, serum calcium, lactate dehydrogenase, serum protein electrophoresis with immunofixation, urine protein electrophoresis with immunofixation, serum-free light chains, and bone marrow examination, along with a radiological skeletal assessment [6].

Management of SP is not well defined. A large study on EMPs which included 1,085 patients comparing head and neck EMPs (HN-EMPs) versus other EMPs showed that HN-EMPs were often localized to the pharynx (21.5%), nasal cavity (19.3%), oral cavity (14.7%), and paranasal sinuses (13.0%). Patients with non-HN-EMPs were more often treated by surgery alone (42% vs. 23,6%; p < 0.001%). On the other hand, HN-EMPs received both surgery and radiation therapy or radiation therapy alone (p < 0.001%). The five-year disease-specific survival (DSS) and five-year overall survival (OS) were better in the HN-EMP group than in other locations. Based on sex, race, stage, and sequence of combination therapy there, were no differences in DSS, but in the HN-EMP group, subcutaneous tissue location had a poorer DSS (p < 0.001%). Surgery alone or combination therapy showed an improvement in 10-year DSS in comparison with radiation therapy alone.

Ozshahin et al. [7] reported a large study of 258 patients with SPs, of whom 214 were treated by radiotherapy alone (as in our case), 34 with chemotherapy and radiotherapy, and eight with surgery alone. The 10-year disease-free survival (DFS) and OS were significantly better for EMP than bone SP. On univariate analysis, age <60 years, extramedullary localization, and tumor size less than 4 cm were good prognostic factors for better OS. On multivariate analysis, radiotherapy was a good prognostic factor for DFS and local control. Bone localization was the only independent predictor for MM development. There was no dose-response relationship for doses more than 30 Gy regardless of tumor size [7]. Nevertheless, another study suggested that tumors measuring more than 5 cm had a worse local control than tumors <5 cm (38% vs. 100%), suggesting that larger tumors need dose escalation to improve local control [8]. A recently published case presented a patient treated for skull base SP with a total dose of 40 Gy with a complete response one year after completion of radiation therapy [9]. To date, there is no consensus on the total dose of radiotherapy for SP, but a dose of 40-50 Gy at 1.8-2.0 Gy per fraction five days a week is generally recommended for the treatment of SP with a margin of 2 cm to encompass the microscopically involved tissue [10].

A meta-analysis that included 46 patients with EMP of the skull base treated with surgery (open and endoscopic) or radiotherapy showed no differences in clear margins achieved by open or endoscopic surgery and a similar survival rate in patients treated with surgery or radiotherapy [11].

Prognostic factors are not well defined, but as reported, bone location is considered a worse prognostic factor, as well as bone marrow infiltration which is associated with a decreased progression-free survival [12]. Although it alters the survival only in patients with bone SP, it also significantly increases the probability of progression to MM in bone SP, without any differences in the case of EMP [13]. However, there are more prognostic factors that predict the progression to MM. Fouquet et al. reported a series of 43 patients and determined that an abnormal serum-free light chain and the presence of two lesions or more (on fludeoxyglucose-positron emission tomography) at the diagnosis shortened the time to progression to MM [14].

## Conclusions

SP, especially in the nasal cavity, is a rare plasma cell disorder. Diagnosis relies on specific criteria from the International Myeloma Working Group, emphasizing biopsy, immunohistochemistry, and comprehensive assessments to distinguish SP from MM. In our case, treatment with radiation therapy led to an initial complete response, but regional recurrence and further bone lesions were detected, which may require more aggressive treatment such as an extended field radiotherapy and/or concurrent chemoradiotherapy. This case underscores the importance of thorough diagnostic workups, vigilant monitoring, and personalized treatment strategies to manage SP and its potential progression to MM. Further research is needed to refine management protocols and improve prognostic outcomes for SP patients.
